# Comparison of Glomerular Transcriptome Profiles of Adult-Onset Steroid Sensitive Focal Segmental Glomerulosclerosis and Minimal Change Disease

**DOI:** 10.1371/journal.pone.0140453

**Published:** 2015-11-04

**Authors:** Jun Tong, Jingyuan Xie, Hong Ren, Jian Liu, Weijia Zhang, Chengguo Wei, Jing Xu, Wen Zhang, Xiao Li, Weiming Wang, Danfeng Lv, John Cijiang He, Nan Chen

**Affiliations:** 1 Department of Nephrology, Ruijin Hospital, Shanghai Jiao Tong University School of Medicine, Shanghai, P. R. China; 2 Institute of Nephrology, Shanghai Jiao Tong University School of Medicine, Shanghai, P. R. China; 3 Department of Medicine, Division of Nephrology, Icahn School of Medicine at Mount Sinai, New York, New York, United States of America; 4 National Center for Gene Research and Institute of Plant Physiology and Ecology, Chinese Academy of Sciences, Shanghai, P. R. China; University of Houston, UNITED STATES

## Abstract

**Objective:**

To search for biomarkers to differentiate primary focal segmental glomerulosclerosis (FSGS) and minimal change disease (MCD).

**Methods:**

We isolated glomeruli from kidney biopsies of 6 patients with adult-onset steroid sensitiveFSGS and 5 patients with MCD, and compared the profiles of glomerular transcriptomes between the two groups of patients using microarray analysis.

**Results:**

Analysis of differential expressed genes (DEGs) revealed that up-regulated DEGs in FSGS patients compared with MCD patients were primarily involved in spermatogenesis, gamete generation, regulation of muscle contraction, response to unfolded protein, cell proliferation and skeletal system development. The down-regulated DEGs were primarily related to metabolic process, intracellular transport, oxidation/reduction andestablishment of intracellular localization. We validated the expression of the top 6 up-regulated and top 6 down-regulated DEGs using real-time PCR. Membrane metallo-endopeptidase (MME) is a down-regulated gene that was previously identified as a key gene for kidney development. Immunostaining confirmed that the protein expression of MME decreased significantly in FSGS kidneys compared with MCD kidneys.

**Conclusions:**

This report was the first study to examine transcriptomes in Chinese patients with various glomerular diseases. Expressions of MME both in RNA and protein level decreased significantly in glomeruli of FSGS kidneys compared with MCD kidneys. Our data suggested that MME might play a role in the normal physiological function of podocytes and a decrease in MME expression might be related to podocyte injury. We also identified genes and pathways specific for FSGS versus MCD, and our data could help identify potential new biomarkers for the differential diagnosis between these two diseases.

## Introduction

Both minimal change disease (MCD) and focal segmental glomerulosclerosis (FSGS) are common causes of nephrotic syndrome in adults and children[1]. The primary treatment strategies of MCD and FSGS include corticosteroids and immunosuppressants[2].Patients with MCD usually achieve complete remission with corticosteroid therapy, but the majority of patients with FSGS manifest as corticosteroid-dependent or resistant[3].Differences in the treatment responses and prognoses support the importance of a diagnostic marker to differentiate between these two diseases.

The diagnosis of FSGS is based on kidney biopsies that show focal and segmental areas of glomerular sclerosis and tuft collapse[4].However, histological diagnosis has limitations because it does not reflect the underlying molecular mechanisms. Additionally, most kidney biopsies are performed in patients with advanced disease.

FSGS is a glomerular disease that is primarily caused by podocyte injury. Therefore, it is important to analyze the transcriptome in isolated glomeruli instead of kidney cortices. The profiling of gene expression in glomeruli could aid the identification of differentially expressed genes between glomerular diseases and define specific molecular subclasses of diseases. Gene expression profiles were reported in glomeruli isolated from biopsy samples of patients with diabetic nephropathy,[5] lupus nephritis,[6] obesity-associated glomerulopathy,[7] and FSGS. Three microarray studies were performed on FSGS patients. Schwab[8] studied the transcriptome characters of childhood-onset FSGS, which might be different from adult FSGS patients. Hodgin[9] used mRNA extracted from formalin-fixed, paraffin-embedded renal specimens, which were not suitable for transcriptome analysis because the RNA integrity and quantity might be injured during the sample preparation. Other studies involved comparatively smaller numbers of patients, making themselves less reliable. For instance, only 4 female FSGS patients were recruited for microarray analysis in Bennett’s study.Glomeruli isolated for the current study were collected from kidney biopsies of 6 FSGS patients and 5 MCD patients. Total RNA was extracted from isolated glomeruli for gene expression profiling tests. Transcriptomic data were analyzed and validated using quantitative PCR and immunohistochemistry to identify differential expressed genes (DEGs) between FSGS and MCD groups. DEGs were further analyzed to determine the potential cellular and biological processes involved in these diseases.

## Materials and Methods

### Ethics Statement

The Institutional Review Board of Ruijin Hospital, Shanghai Jiao Tong University School of Medicine approved this study, which was performed according to the Principles in the Helsinki Declaration II. Written informed consent was obtained from each patient.

All renal biopsies were performed by percutaneous technique using standard ultrasound imaging instruments (Diagnostic ultrasound system Aplio Model SSA-770A; Toshiba Medical Systems Corporation, Japan) and a 15 cm and a 12 cm long needle with penetration depth ranged from 3.5 to 5.5 cm and sample notch from 3.5 to 4 cm kidney tissues were originally collected for pathological diagnosis including standard procedures of light, immunofluorescence and electron microscopy.

After the above process, additional kidney tissues were archived and were cut into slices less than 0.5 cm thick using a scalpel, placed into a Rnase free freezing tube, then stored in liquid nitrogen.

### Patients

All of the patients who were recruited in this study were admitted and followed up in the Nephrology Department in Ruijin Hospital, Shanghai Jiao Tong University School of Medicine. We prospectively collected kidney biopsies from patients with newly diagnosed nephrotic syndrome (defined as 24 hour urinary protein > 3.5 g/d, serum albumin < 30g/L, referred to 2012 Kidney Disease: Improving Global Outcomes (KDIGO) clinical practice guideline on glomerulonephritis) when receiving renal biopsy. Subsequently, patients with nephrotic syndrome were selected who were responded to prednisone treatment (1mg/kg/d) within 6–8 weeks dated from the recruitment.

**Fig 1:**
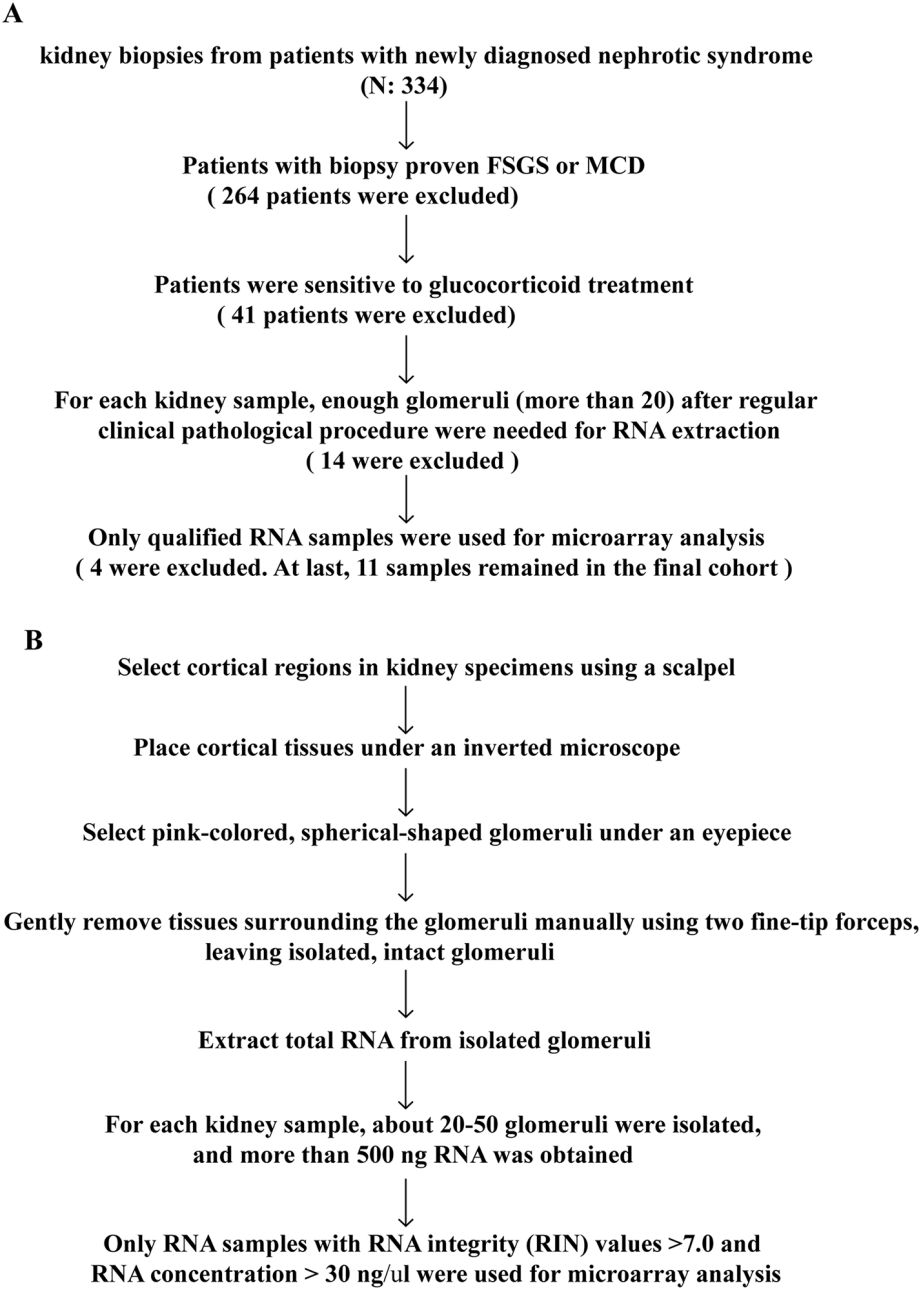
Inclusion criteria and experimental workflow. A, Inclusion criteria of patient selection; B, Experimental workflow of glomerulus isolation.

The inclusion criteria of patient selection and procedures of glomerulus isolation were presented in Fig 1A and 1B respectively, an informed consent was signed by the patient. The following exclusion criteria were used: 1) Obesity(BMI < 30); 2) HIV-associated nephropathy; 3) Infection; 4) Reflux nephropathy; 5) Autoimmune diseases; 6) Malignant cancers; 7) Alcoholism or long-term smoker (more than 3 months); or 8) Patients had a family history of kidney diseases or extrarenal manifestations, including hearing loss and eye problems that are suggestive of hereditary kidney diseases, such as Alport's syndrome (AS), thin basement membrane disease (TBMD) or Fabry disease. The diagnosis and differentiation of CKD stage were determined according to the criteria of the National Kidney Foundation (NKF). The glomerular filtration rate (GFR) was estimated using the equation from the study ‘‘Modification of Diet in Renal Disease” (MDRD)[10].

We recruited MCD patients as the control group. To avoid the bias originated from response to steroid therapy, in the current study, we only recruited patients with steroid-sensitive nephrotic syndrome both in FSGS and MCD groups.

Clinical characteristics were recorded for all patients at baseline and during follow-up. Two senior pathologists reviewed all renal biopsy slides. A semiquantitative scoring method was adopted to evaluate pathological lesions. A percentage of focal and global glomerulosclerosis was recorded, and a score of 0 (0%), 1 (<25%), 2 (25–50%) or 3 (>50%) was assigned to each slide to reflect the severity of tubular atrophy, interstitial fibrosis, and inflammatory cell infiltration[11].

### Glomerulus Isolation and RNA Extraction

Cortical regions in kidney specimens were selected using a scalpel, and tissues were placed on an ice bath under an inverted microscope. Pink-colored, spherical-shaped glomeruli were detected under an eyepiece, and isolated for RNA extraction. Procedures of glomerulus isolation were presented as a flowchart (Fig 1B).

Total RNA of isolated glomeruli was prepared using the Ambion RNAqueous^®^-Micro Kit (Ambion, AM1931, USA) according to the manufacturer’s protocol. All of the RNA samples were analyzed using a Bioanalyzer (2100 RNA Quality Control, Agilent Technologies) to verify sample purity. RNA concentrations were determined, using a NanoDrop 2000 (Thermo, Wilmington, USA) at an absorbance of 260 nm, and quality control standards were A260/A280 = 1.8–2.1. Only RNA samples with RNA integrity (RIN) values > 7.0 and an RNA concentration > 30 ng/μl were used for microarray analysis. For each kidney sample, about 20–50 glomeruli were isolated, and more than 500 ng qualified RNA was obtained.

### Microarray Hybridization

Total RNA was converted into cDNA, which was fragmented, labeled, and hybridized onto gene chips (Microarray Gene 1.0, Affymetrix, Santa Clara, CA) according to Affymetrix standard protocols. Affymetrix^®^ Expression Console Software (version 1.2.1) was used for microarray analyses.

Expression data of all probesets detected by microarray analysis have been deposited in Gene Expression omnibus, GPL6244GSE, GSE69814.

### Analysis of Microarray Data

Robust Multichip Average (RMA) was used with the default configuration for background adjustment and normalization[12]. LIMMA was used using a q-value < 5% for the identification of differentially expressed genes in microarray experiments in R[13].The threshold was set to P <0.005 for LIMMA and fold-change > 1.5. PCA analysis was performed to assess sample correlations using the expression data of all detected genes. Functional analyses (Gene Ontology, Pathway analysis) of DEGs were performed using the online tool Enrichr program[14].

### Validation with Real-time PCR

Twelve gene transcripts, including the top 6 up-regulated genes (SNORA38B, RN5S451, RN5S421, LOC728419, SNORA14A, and SNORA7B) and top 6 down-regulated genes (CALB1, GSTA1, MME, AK4, ANGPTL3, and PLG) in FSGS compared with MCD were chosen for validation. The mRNA expression levels of these genes was confirmed using real-time PCR from fourteen additional RNA samples from patients with histologically diagnosed FSGS or MCD who manifested with newly diagnosed nephrotic syndrome without previously using glucocorticoids or immunosuppressants. These patients were distinct from the patients we selected for microarray analysis. The clinical parameter, treatment strategy and outcome of patients were presented in Tables A-D in S1 File. First-strand cDNA was prepared from total RNA samples (0.5 μg) using the SuperScript^TM^ III First-Strand Synthesis Kit (Invitrogen), and cDNA (1 μl) was amplified in triplicate using the SYBR GreenER qPCR Supermix in an ABI PRISM 7900 HT (Applied Biosystems, Foster City, CA). Primers were designed using PrimerBlast (http://www.ncbi.nlm.nih.gov/tools/primer-blast/, last accessed February 11, 2015) and synthesized by Sigma. Light cycler analysis software was used to determine crossing points using the second derivative method. Data were normalized to the housekeeping gene GAPDH, and results are presented as fold-changes between different groups using the Pfaffl method[15].

### Immunohistochemistry

Archival corresponding patient biopsy specimens were collected from Ruijin Hospital under a procedure approved by its Institutional Review Board. 14 additional biopsy samples have been used for the MME immunostaining, including 7 samples obtained from FSGS patients, 7 samples from MCD patients. Specimens were baked for 20 minutes at 55–60°C in an oven and processed as follows. Formalin-fixed and paraffin-embedded specimens were deparaffinized, and H_2_O_2_ was used to inactivate endogenous peroxidase. Specimens were blocked in 2% goat serum diluted in phosphate-buffered saline (PBS) for 1 hour at room temperature and incubated in a 1:50-diluted mouse anti-MME antibody (Thermo, Grand Island, NY) at 4°C overnight. Subsequently, specimens were washed three times with PBS and incubated in a 1:1000-diluted secondary antibody (goat anti mouse IgG, Thermo, Grand Island, NY) for 2 hours at room temperature. Positive staining was revealed using peroxidase-labeled streptavidin and a diaminobenzidine substrate[16]. For determination of immunohistochemistry staining, stained sections were imaged using the Image Analysis System (AxioVision 4, Carl Zeiss, Germany). The positively stained cells in the glomeruli were counted from six randomized selected areas of kidney sections for each patient, and expressed as the number of positively stained cells per square millimeter of glomerular cross-section, then the difference was analyzed by one-way ANOVA followed by Bonferroni correction. A P value less than 0.05 was considered statistically significant.

### Statistical analysis

Distributions for categorical variables are described as frequencies and percentages, and proportions between groups were compared using a χ^2^ test. Distributions for normally distributed quantitative variables are described as the arithmetic means and standard deviations (or medians and ranges for non-normally distributed variables). Student's *t*-test or Mann-Whitney U test was used to compare the continued variables based on their distributions. In immunohistochemical analysis, the difference among varied groups was analyzed by one-way ANOVA followed by Bonferroni’s post hoc test. A P—value less than 0.05 was considered statistically significant.

## Results

### Clinical Parameters

The clinical parameter and outcome of patients enrolled in the microarray analysis were recorded (Tables E-H in S1 File). Patients receive 1mg/kg/d prednisone treatment after diagnosis, accroding to 2012 Kidney Disease: Improving Global Outcomes (KDIGO) clinical practice guideline on glomerulonephritis (GN).The mean follow-up time was 13.67±12.51 months for the FSGS group and 12.8±7.40 months for the MCD group (p > 0.05). Eleven patients with nephrotic syndrome (FSGS, n = 6; MCD, n = 5) were enrolled in our experiment. Four FSGS patients (F1, F2, F4, F5) and 4 MCD patients (M1, M2, M3, M5) were at CKD1 (72.73%). One MCD patient (M4) was at CKD2 (9.09%), and two FSGS patients (F3, F6) were at CKD3 (18.18%). No significant differences in baseline clinical parameters were found between the two groups, except a higher systolic blood pressure in the FSGS patients (Table 1).

**Table 1:** Clinical Parameters of Patients with FSGS and MCD.

	FSGS	MCD	*P* value
**Number**	6	5	
**Male/Female**	4/2	3/2	0.699
**Follow up, months**	9(4.05–23.28)	13(3.62–21.98)	0.699
**Height, cm**	167.11±7.88	163.2±9.73	0.428
**Weight, kg**	66.5±15.80	55.5±9.66	0.186
**BMI**	23.78±5.36	20.76±2.46	0.261
**SBP, mmHg**	128(122.30–163.03)	118(98.23–132.17)	0.042*
**DBP, mmHg**	85(77.22–96.55)	74(61.56–92.04)	0.18
**Age of onset**	31.78±15.31	22.2±7.69	0.22
**Serum creatinine, μmol/L**	91±49.16	70.8±19.8	0.403
**Uric acid, μmol/L**	349.67±112.01	337.6±89.24	0.84
**eGFR, ml/min per 1.73 m** ^**2**^	99.92(69.34–128.13)	121.74(71.16–179.5)	0.364
**Serum total protein, g/L**	40.78±8.53	34.6±7.40	0.2
**Serum albumin, g/L**	17.67±7.76	12.4±5.32	0.205
**24 hr UprV, g**	10.93(5.95–17.31)	5.99(0.46–12.76)	0.378
**ACR, mg/mmol**	625.10(431.68–1171.24)	473.1(236.08–655.56)	0.206
**Hemoglobin, g/L**	139.40±16.89	147.40±12.36	0.418
**Hct**	0.41±0.05	0.43±0.03	0.431
**Fast blood glucose, mmol/L**	4.08±0.78	4.25±0.64	0.718
**2 hour post-meal blood glucose, mmol/L**	5.52±1.03	5.12±1.07	0.56
**TC, mmol/L**	9.20±2.38	10.20±2.12	0.481
**TG, mmol/L**	2.59±0.93	3.41±1.25	0.271
**HDL, mmol/L**	1.33±0.46	1.47±0.44	0.644
**LDL, mmol/L**	6.69±1.95	7.36±1.99	0.606
**Lp(a), mmol/L**	0.73±0.38	0.73±0.37	0.983
**focal glomerulosclerosis,%**	10.29 (6.25–28.80)	0	0.023*
**global glomerulosclerosis,%**	19±12.7	1±0.6	0.205
**adhesion of capillary loop,%**	6(2–13)	0	0.024*
**Tubulointerstitial tissue lesion score**	4.33±4.16	2.17±0.98	0.238

BMI: body mass index; SBP: systolic blood pressure; DBP: diastolic blood pressure; GFR: glomerular filtration rate; Alb: albumin; 24 hr UprV: protein amount of 24 hours urine; ACR: albumin-creatinine ratio; Hct: Hematocrit; TC: total cholesterol; TG: triglycerides; HDL: high density lipoprotein; LDL: low density lipoprotein; Lp(a): Lipoprotein (a).

*P< 0.05, FSGS versus MCD patients.

### Microarray Analysis

**Fig 2:**
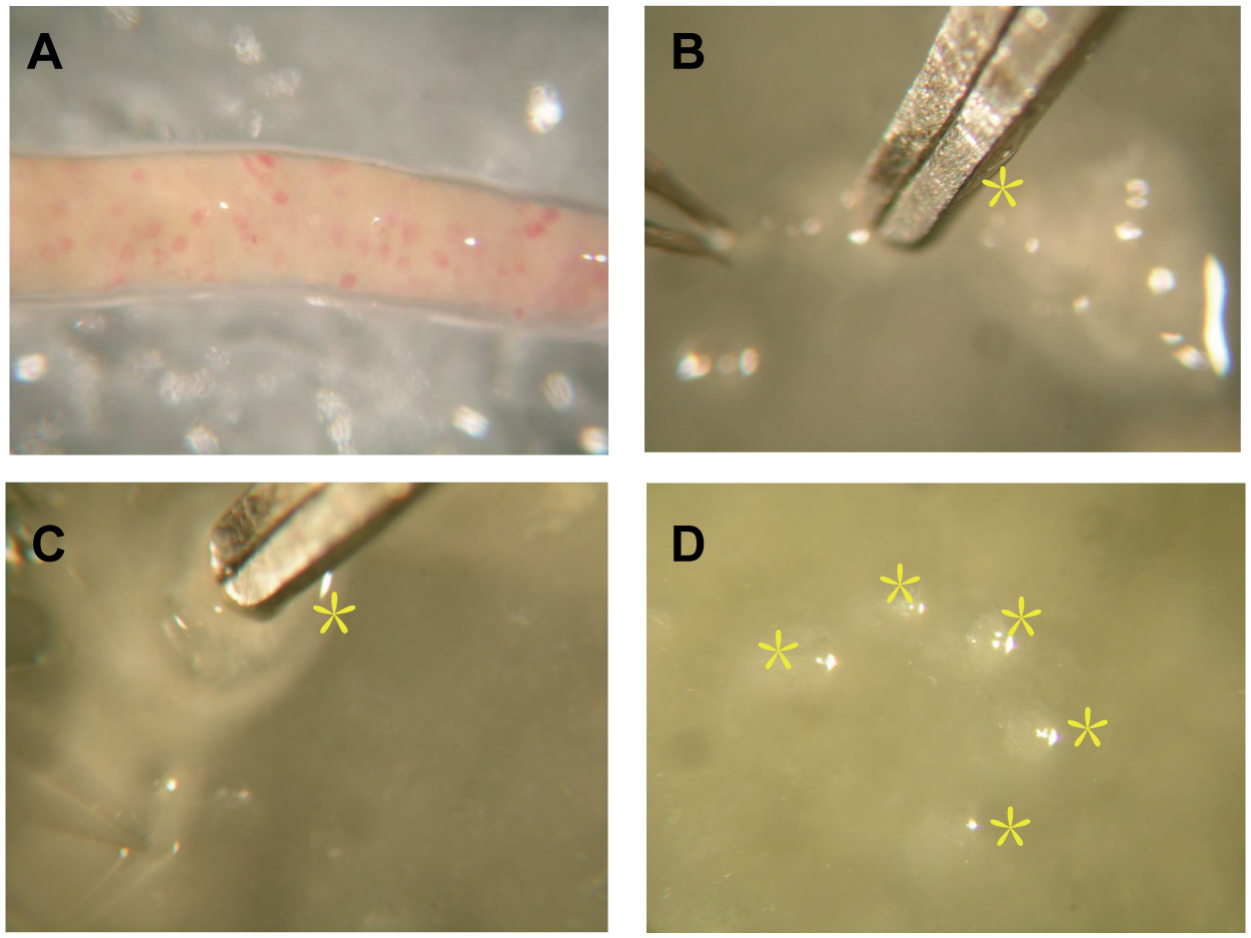
Glomerulus isolation. A, Cortex of kidney biopsy; B, Glomerulus isolation under an inverted microscope; C, Isolated glomerulus; D, 5 isolated glomerulus were pulled together.

**Fig 3:**
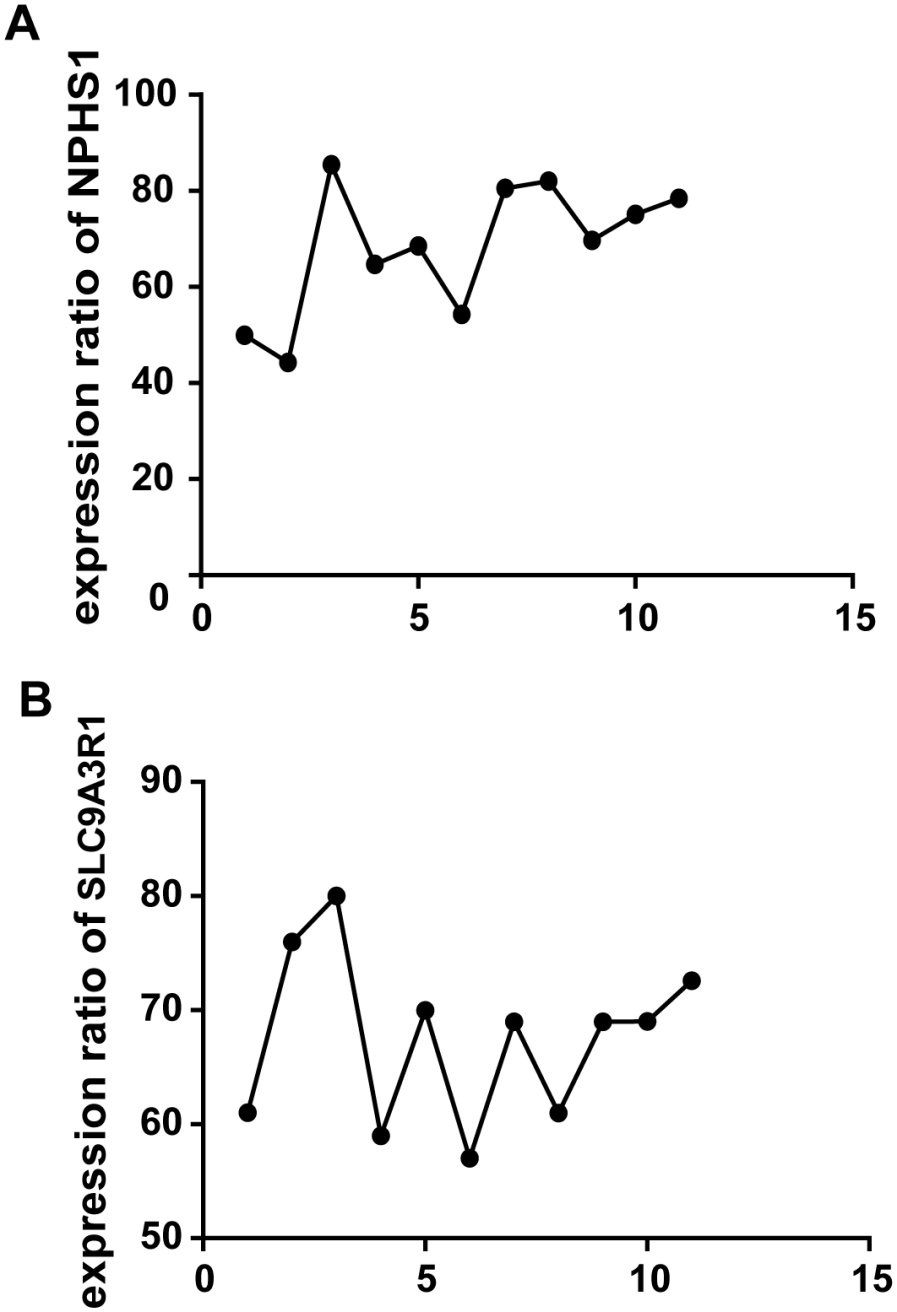
Comparision of expression ratio of NPHS1, SLC9A3R1 in glomerulus versus tubulointerstitial using real-time PCR. A, Comparision of expression ratio of NPHS1 in isolated glomerulus versus tubulointerstitial; B, Comparision of expression ratio of SLC9A3R1 in tubulointerstitial versus glomerulus (Difference was analysed by Student's *t*-test, ** indicated *P* < 0.01).

We isolated glomeruli from archived kidney tissue, the process of isolating glomeruli under an inverted microscope was presented in Fig 2. We verified the expression ratio of NPHS1, SLC9A3R1 in glomeruli and tubulointerstitial. NPHS1 is a podocyte marker, which made it a glomerular marker, and the expression ratio of NPHS1 in glomeruli versus tubulointerstitial was 70.14, Fig 3A; SLC9A3R1 is a tubular epithelium marker, and the expression ratio of SLC9A3R1 in tubulointerstitial versus glomeruli was 69.42, Fig 3B. Therefore, we isolated glomeruli without contamination of tubulointerstitial.

**Fig 4:**
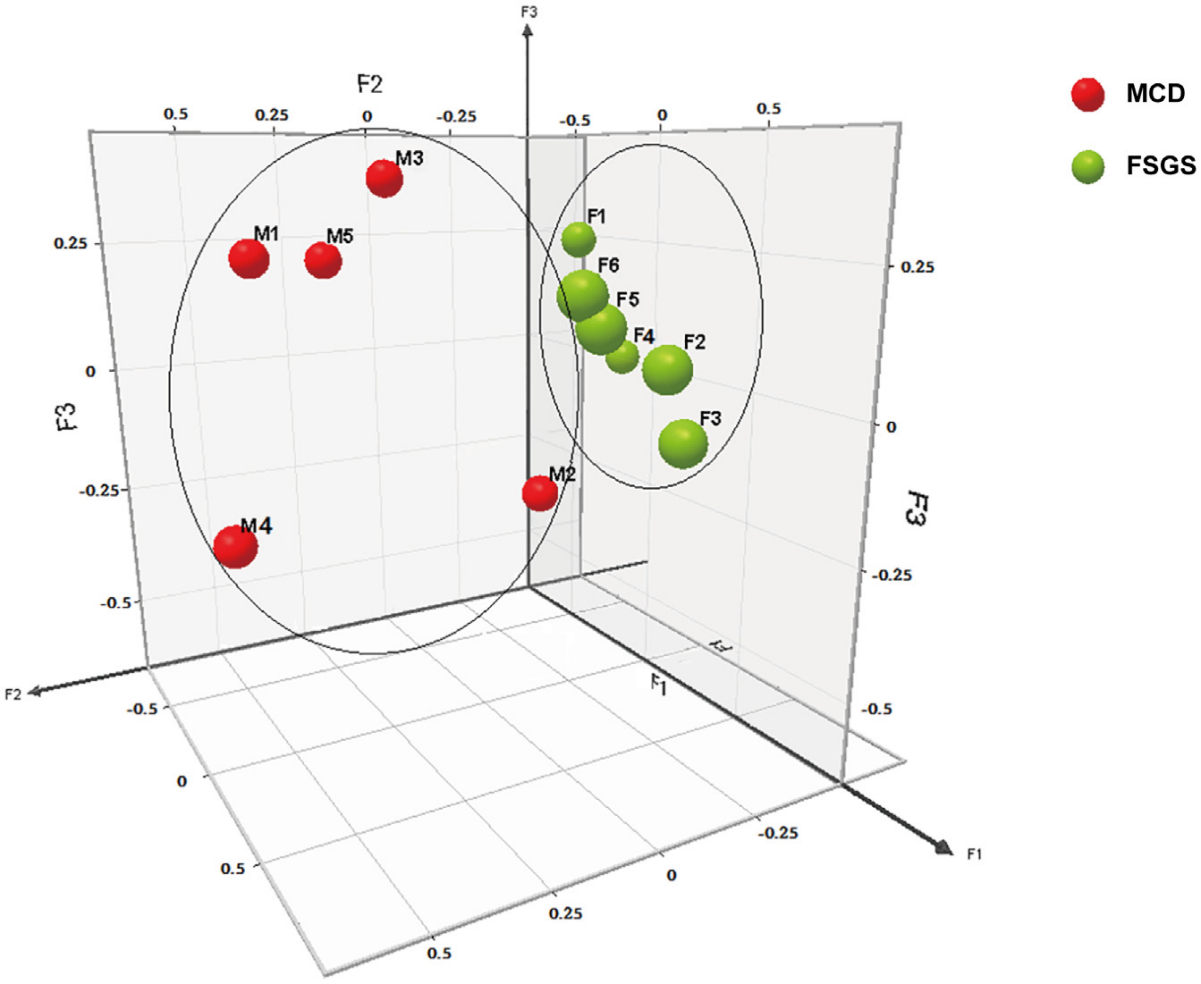
3D snapshot of PCA analysis of sample distribution based on the transcriptomes. The FSGS group was separated from the MCD group. Moreover, FSGS patients were far from MCD patients, which indicated dramatic transcriptomic changes in FSGS patients compared with MCD patients. F1 ~ F6 (green sphere) indicated FSGS patients; M1 ~ M5 (red sphere) indicated MCD patients.

**Fig 5:**
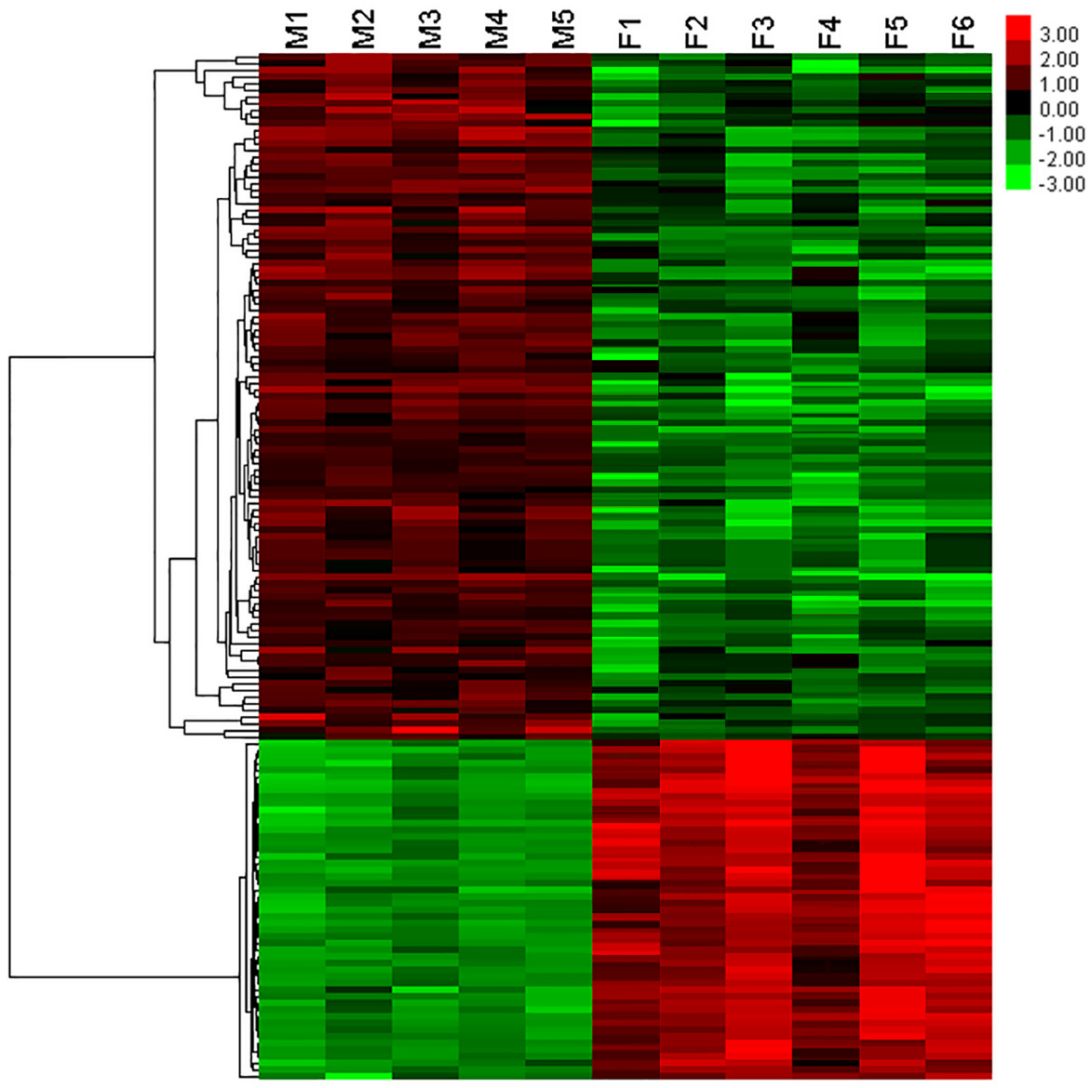
The Heat Map of gene expression profiles between FSGS and MCD patients. A total of 272 genes were up-regulated; A total of 2331 genes were down-regulated. Red indicated high expression. Green indicated low expression. Black indicated no significant difference between FSGS and MCD patients. F1 ~ F6 indicated FSGS patients. M1 ~ M6 indicated MCD patients.

We performed microarray analysis of glomerular gene expression from FSGS and MCD patients and identified DEGs between these two groups. We performed PCA analyses to identify sample correlations using the raw data obtained from microarray studies (Fig 4). Comparisons of the gene expression profiles between FSGS and MCD patients revealed several patterns of disease-specific changes between the two diseases (Fig 5). Gene ontology and pathway analyses were also performed in the DEGs.

**Fig 6:**
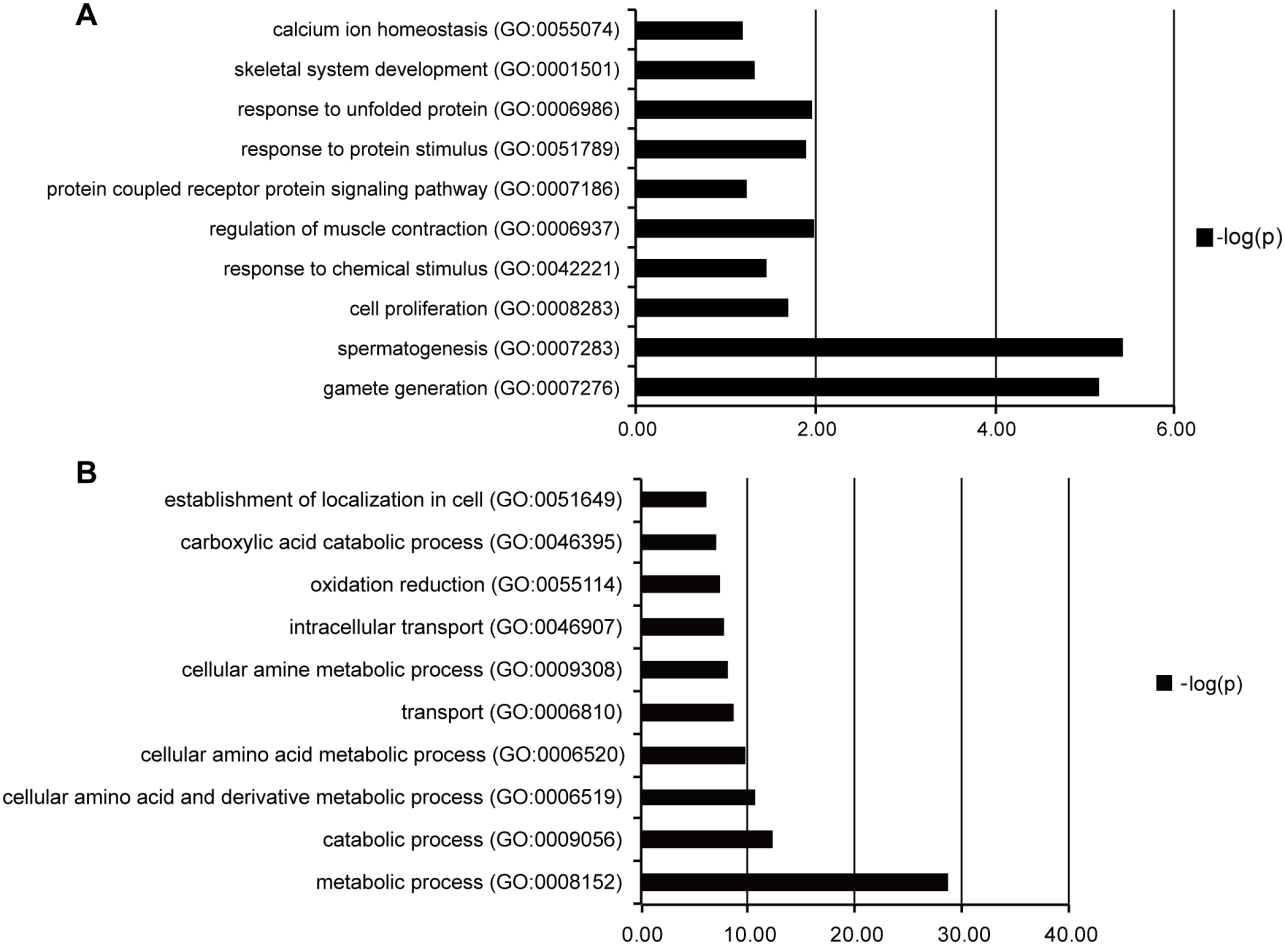
Pathway of genes up-regulated and down-regulated in FSGS patients compared with MCD patients analyzed by using Enrichr GO Biological Process program. A: Pathway of genes up-regulated; B: Pathway of genes down-regulated.

Among DEGs, 272 genes were up-regulated in FSGS compared with MCD. These genes are involved in spermatogenesis, gamete generation, regulation of muscle contraction, response to unfolded protein, cell proliferation and skeletal system development. A total of 2331 genes were down-regulated, and these genes are related to metabolic process, intracellular transport, oxidation reduction, establishment of localization in cell bodies (Fig 6). The large number of genes were deposited in the supplemental material. (Tables I–L in S2 File, Table M in S3 File).

### Validation of Gene Expression using Real-time PCR

**Fig 7:**
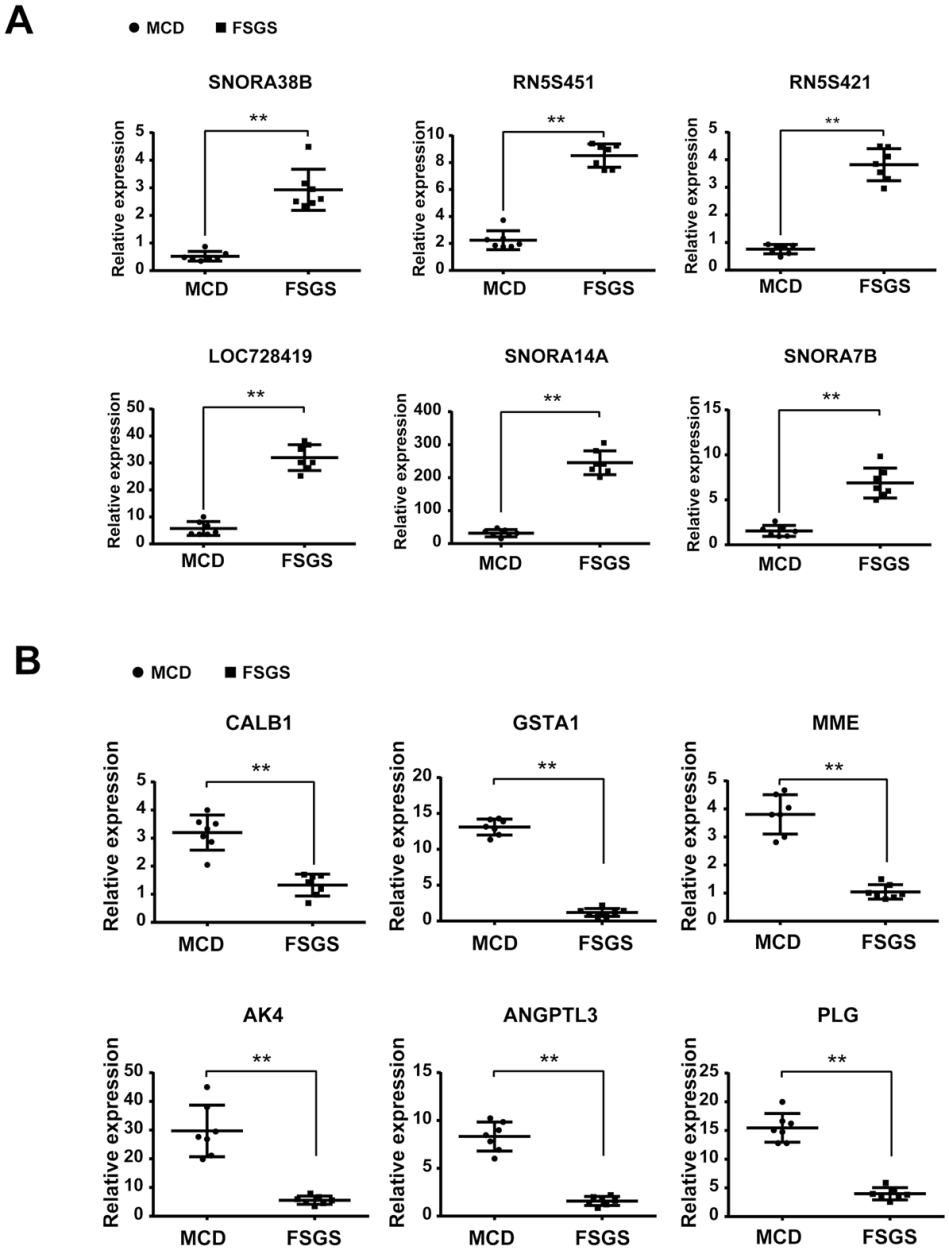
Validation of the top 6 genes up-regulated and the top 6 genes down-regulated in FSGS patients compared with MCD patients using real-time PCR in glomerular transcriptomes. (Student's *t*-test or Mann-Whitney U test was used to compare the continued variables based on their distributions.**P < 0.01, FSGS patients compared with MCD patients). A, Validation of the top 6 genes up-regulated; B, Validation of the top 6 genes down-regulated.

**Fig 8:**
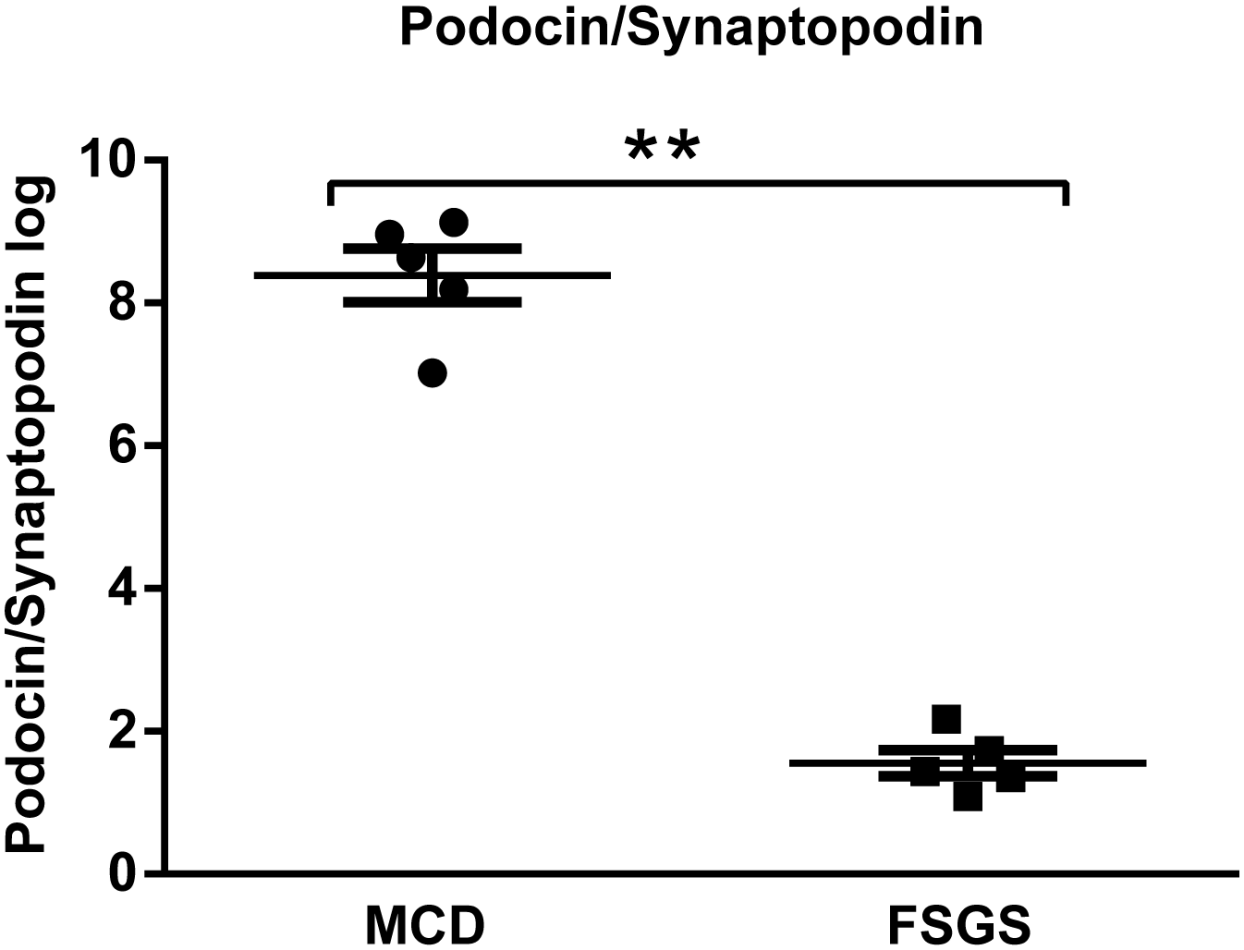
Validation of podocin/synaptopodin mRNA expression ratio in isolated glomeruli between MCD and FSGS patients using real-time PCR in glomerular transcriptomes. (Student's *t*-test was used to compare the the podocin/synaptopodin mRNA expression ratios.** *P* < 0.01, FSGS patients compared with MCD patients).

We chose the top 6 up-regulated genes (SNORA38B, RN5S451, RN5S421, LOC728419, SNORA14A, and SNORA7B) and the top 6 down-regulated genes (CALB1, GSTA1, MME, AK4, ANGPTL3, and PLG) in FSGS compared with those in MCD for further validation. Validation was conducted using real-time PCR in an independent cohort composed of 7 FSGS patients and 7 MCD patients. Primer sequences were listed in Table 2. We confirmed that the changes in gene expression in FSGS patients were consistent with the microarray results (Fig 7). Schmid et al[17] have described glomerular podocin/synaptopodin mRNA expression as a potential marker to differentiate between MCD and FSGS, as well as steroid resistent and steroid sensitive cases. Hence, we validated the ratio glomerular podocin/synaptopodin mRNA, and found the ratio of podocin relative to synaptopodin mRNA allowed a clear separation between MCD and FSGS with no overlap (MCD mean ratio, 8.39 ±0.38; FSGS mean ratio, 1.56±0.18; P < 0.01. Fig 8).

**Table 2:** Primer Sequences.

Gene	Forward	Reverse
***SNORA38B***	CCTCCTACAAAGGCATGTCTAT	TTCTATGTGGGATGGTTGATCTT
***RN5S451***	GCCTGCTGCCATAGTACTCTG	CACGTATTCCTACCCAACTTTCTC
***RN5S421***	CCTTGGCAGGCACTGGT	AGCCTCCAGCTCCCAGTCT
***LOC728419***	CAGCTCAGAGTGTCCAGCAA	AGTTAACGTCTTGGAGGCCG
***SNORA14A***	TGCATTCTTAAACCCTCTTGG	AGATGTTGCAGGTATGAAATAAGA
***SNORA7B***	GACCTCCTGGGATCGCAT	CACTGTCGCAGAGTGTCTTCC
***CALB1***	GCTGAGCTTTTGCTCACTCC	ACTTCCGTCAGCGTCGAAAT
***GSTA1***	TGATCCTCCTTCTGCCCGTA	ACCAGATGAATGTCAGCCCG
***MME***	TCTGCTGAGGGGTCACGATT	AGGACCGAGAGGCTGATCTC
***AK4***	CTTTGAGTCACCCCCGCTT	GCCGCCCCTTCATCCTTAAC
***ANGPTL3***	CAATGTCCCCAATGCAATCCC	CCAGCCTCCTGAATAACCCT
***PLG***	TGGGGAGAAACCCAAGGTACT	CACAGAGTTCGGTGGATTGGA
***GAPDH***	GGTGAAGGTCGGAGTCAAC	CAAATGAGCCCCAGCCTTC

### Immunostaining of MME

**Fig 9:**
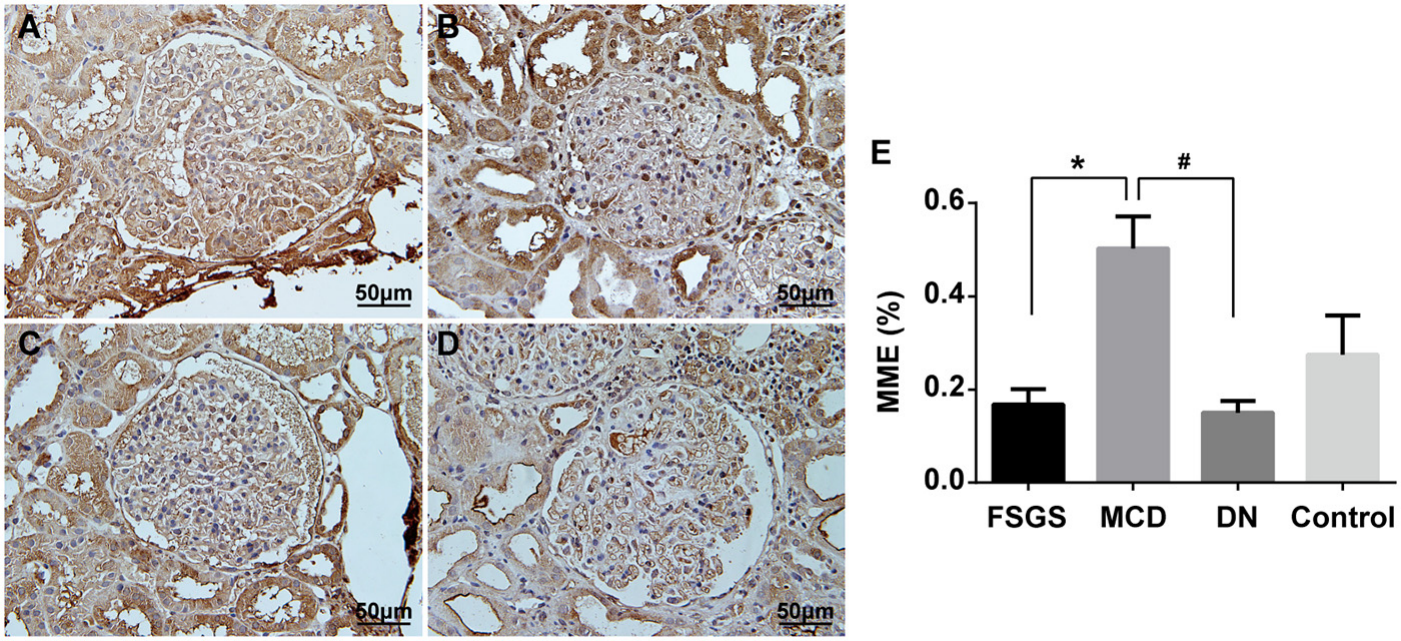
Immunohistochemical staining of MME in kidney biopsies from patients with FSGS, MCD and DN. Representative pictures from individual patients were shown. A:Kidney biopsies from patients with FSGS. B:Kidney biopsies from patients with MCD. C:Kidney biopsies from patients with DN. D:Kidney biopsies from uninvolved portions of a kidney at the time of nephrectomy for renal clear cell carcinoma, as the control. E: Histogram. *P < 0.01, FSGS patients compared with MCD patients, ^#^ *P* < 0.01, DN patients compared with MCD patients.

MME plays an important role in glomerular diseases. Therefore, we selected MME to further validate the expression differences between FSGS and MCD at the protein level using immunostaining. Validation was conducted in an independent cohort composed of 7 FSGS patients and 7 MCD patients. These patients came from the group where the real-time PCR samples were obtained. Protein levels of MME were markedly reduced in kidneys of patients with FSGS compared with patients with MCD (Fig 9). Previous studies suggested that MME was down-regulated in diabetic nephropathy (DN)[18]. Therefore, we also examined MME expression in DN and found that MME expression was decreased in kidneys of patients with DN compared with that in patients with MCD. These findings suggested that MME could be used as a marker to differentiate FSGS from MCD.

## Discussion

We revealed differences in the transcriptional profile between FSGS and MCD using high throughput gene expression profiling of isolated glomeruli from patients with biopsy-proven idiopathic FSGS and MCD. Hodgin et al reported that genes that participated in cell motility, migration, differentiation and morphogenesis were up-regulated in FSGS patients, while podocyte specific genes were significantly down-regulated in FSGS group compared with normal and MCD groups. We found podocyte specific genes (SYNPO, NPHS1) were down-regulated in glomeruli of FSGS patients compared with those of MCD patients, which is consistent with findings from Hodgin et al. Bennett et al[19] reported that genes implicated in kidney fibrosis, the TGF-β signaling pathway, transcription factors that drive chondrogenesis and fibrosis, were up-regulated in FSGS patients. We discovered genes that participated in TGF- β signaling and kidney fibrosis were up-regulated in glomeruli of FSGS patients as opposed to those of MCD patients, which was congruent with findings from Bennett et al. Schwab concluded that genes involved in cell cycle and proliferation, immune responses, TGF- β superfamily signaling, and RNA processing or splicing were up-regulated in kidneys of FSGS patients. We found genes involved in cell cycle and cell proliferation were up-regulated in glomeruli of FSGS patients in contrast to those of MCD patients, which was in line with resultsfrom Schwab et al. However, only Bennett and Hodgin’s studies used mRNA obtained from glomeruli. Schwab using the mRNA obtained from biopsied kidney.

We identified previously unreported up-regulated genes involved in gamete generation, regulation of muscle contraction, response to unfolded protein, cell proliferation in FSGS patients, and down-regulated genes that were mostly related to intracellular transport, oxidation reduction and establishment of localization. Many of these pathways are involved in kidney diseases[20] (Table N in S3 File).

Our data suggested that pathways that were activated in FSGS were quite different from those in MCD. For example, inflammation and fibrosis pathways were more activated in FSGS, and cell cytoskeleton-related pathways were suppressed in FSGS compared with MCD. A significant portion of these genes were identified previously in the pathogenesis of kidney diseases. Podocyte injury played a central role in the pathogenesis of FSGS[21].Several DEGs, such as MME and ANGPTL3, were important for podocyte morphology and function.

MME was involved in kidney development, and it is abundantly expressed in the kidney[22], particularly in podocytes[23]. Expression of MME at mRNA levels was significantly down-regulated in kidneys of patients with diabetic nephropathy (DN) [24]. MME appeared to have excessive activity induced by hyperglycemia, hypertension, and hyperlipidemia related to diabetes[25]. Inhibition of angiotensin-converting enzyme (ACE) is among one of the most effective treatments for hypertension and end-organ damage associated with diabetic nephropathy. Angiotensin-converting enzyme (ACE) inhibitor attenuated Ang II-induced extracellular matrix synthesis more efficiently in the absence of NEP[26]. And MME had a genetic linkage region for DN. MME was also a podocytic antigen that was responsible for human membranous nephropathy (MN). Alloimmunization against MME should be considered as a leading cause of membranous glomerulopathy early in life[27]. Absence of MME gene product in the mother resulted in the development of membranous nephropathy in the fetus because maternal anti-MME antibodies bound to MME on fetal podocytes[28]. The fetal podocytes undergoing apoptosis and nephron loss could lead to chronic renal failure in early adulthood.

Notably, our data demonstrated that MME might play a protective role in the normal physiological function of podocytes and a decrease in MME expression might cause podocyte injury, which leads to FSGS. However, there was no report about the role of MME in FSGS, and further studies were required to validate our results.

Among the top genes identified in our study, SNORA38B, SNORA14A and SNORA7B were small nucleolar RNA that function as ribonucleoprotein (RNP) enzymes in the processing of ribosomal RNAs (rRNAs) and small nuclear RNAs (snRNAs). The actions of these enzymes were related to mRNA splicing, genome integrity maintenance, and protein synthesis[29]. RN5S451 and RN5S421 were 5S rRNAs, which were major components of the fully functional ribosome that was responsible for protein synthesis[30]. LOC728419 (ubiquitin carboxyl-terminal hydrolase 17-like) was involved in ubiquitin-dependent apoptotic process[31].CALB1 (calbindin 1) primarily functions in metanephric ureteric bud development[32]. GSTA1 (glutathione S-transferase alpha 1) was a key enzyme in glutathione and xenobiotic metabolic processes and the generation of oxidative products[33]. AK4 (adenylate kinase 4) was responsible for cell proliferation, differentiation and cytoskeleton formation[34]. AK4 catalyzed the reversible transfer of adenosine triphosphate (ATP) or guanosine triphosphate (GTP) to adenosine monophosphate (AMP), and it played a key role in high-energy phosphoryl transfer and consumption of ATP and GTP[35]. AK4 was also an essential enzyme in energy metabolism in the cytosol, mitochondria and nucleus. ANGPTL3 (angiopoietin-like 3) was responsible for angiogenesis, glycerol, fatty acid metabolic processes, cell matrix adhesion, and integrin-mediated signaling pathways, and a role for ANGPTL3 in podocyte injury and glomerular disease was shown recently. Deletion of ANGPTL3 or interfering with the ANGPTL3-integrin β3 interaction might be benefit for podocyte protection and attenuates proteinuria[36]. PLG (plasminogen) was an enzyme that degraded fibrin clots and participates in apoptotic processes. Future studies were required to determine the function of these genes in FSGS and whether these genes might serve as biomarkers for the differential diagnosis of FSGS and MCD.

Limitations of our study included the relatively small sample size and the absence of normal kidney specimens as a control group. The nephrectomy samples might not be a good control as the ischemic changes between biopsy and nephrectomy samples were quite different. Therefore, we compared samples between FSGS and MCD patients. Future studies, such as the studies proposed in NEPTUNE, were required to determine gene expression profiles in a large patient population to validate our findings. In addition, our study has limited power to detect baseline characteristic differences between patients with MCD and FSGS. However, our study is the first transcriptomic analysis based on glomeruli between FSGS and MCD patients in the Chinese population. Our data suggested that this approach may reveal the underlying molecular mechanisms of FSGS and MCD and identify potential biomarkers to aid the differential diagnosis between these two diseases.

## Conclusions

In conclusion, we identified the up-regulated DEGs in FSGS patients compared with MCD patients were primarily involved in spermatogenesis, gamete generation, regulation of muscle contraction, response to unfolded protein, cell proliferation, skeletal system development. The down-regulated DEGs were primarily related to metabolic process, intracellular transport, oxidation reduction, establishment of localization in cell. Among these genes, MME was a down-regulated gene that was previously identified as a candidate gene for kidney development. Expression of MME both in RNA and protein levels were decreased significantly in glomeruli of FSGS kidneys compared with MCD kidneys.

## Supporting Information

S1 File **Table A**- Clinical and pathological parameter of patients with FSGS enrolled in real-time PCR analysis; **Table B**- Treatment strategy and outcome of patients with FSGS enrolled in real-time PCR analysis; **Table C**- Clinical and pathological parameter of patients with MCD enrolled in real-time PCR analysis; **Table D**- Treatment strategy and outcome of patients with MCD enrolled in real-time PCR analysis; **Table E**- Clinical and pathological parameter of patients with FSGS enrolled in microarray analysis; **Table F**- Treatment strategy and outcome of patients with FSGS enrolled in microarray analysis; **Table G**- Clinical and pathological parameter of patients with MCD enrolled in microarray analysis; **Table H**- Treatment strategy and outcome of patients with MCD enrolled in microarray analysis.
(XLSX)

S2 File **Table I-** The 272 genes, which were up-regulated in FSGS patients compared with MCD patients, were analyzed by using Enrichr KEGG program; **Table J-** The 2331 genes, which were down-regulated in FSGS patients compared with MCD patients, were analyzed by using Enrichr KEGG program; **Table K-** The 272 genes, which were up-regulated in FSGS patients compared with MCD patients, were analyzed by using Enrichr GO Biological Process program; **Table L-** The 2331 genes, which were down-regulated in FSGS patients compared with MCD patients, were further analyzed by using Enrichr GO Biological Process program.
(XLSX)

S3 File **Table M-** All probesets detected by microarray analysis; **Table N-** Compare the current data to those of previous reports.
(XLSX)
